# Restoring Homeostasis: Treating Amyotrophic Lateral Sclerosis by Resolving Dynamic Regulatory Instability

**DOI:** 10.3390/ijms26030872

**Published:** 2025-01-21

**Authors:** Albert J. B. Lee, Sarah Bi, Eleanor Ridgeway, Irfan Al-Hussaini, Sakshi Deshpande, Adam Krueger, Ahad Khatri, Dennis Tsui, Jennifer Deng, Cassie S. Mitchell

**Affiliations:** 1 Laboratory for Pathology Dynamics, Department of Biomedical Engineering, Georgia Institute of Technology and Emory University, Atlanta, GA 30332, USA; 2 Center for Machine Learning at Georgia Tech, Georgia Institute of Technology, Atlanta, GA 30332, USA

**Keywords:** amyotrophic lateral sclerosis, SOD1 transgenic mouse model, neurodegeneration, pathology dynamics, homeostasis, motor neuron disease, neuromuscular, first-order feedback control system, systems biology

## Abstract

Amyotrophic lateral sclerosis (ALS) has an interactive, multifactorial etiology that makes treatment success elusive. This study evaluates how regulatory dynamics impact disease progression and treatment. Computational models of wild-type (WT) and transgenic SOD1-G93A mouse physiology dynamics were built using the first-principles-based first-order feedback framework of dynamic meta-analysis with parameter optimization. Two in silico models were developed: a WT mouse model to simulate normal homeostasis and a SOD1-G93A ALS model to simulate ALS pathology dynamics and their response to in silico treatments. The model simulates functional molecular mechanisms for apoptosis, metal chelation, energetics, excitotoxicity, inflammation, oxidative stress, and proteomics using curated data from published SOD1-G93A mouse experiments. Temporal disease progression measures (rotarod, grip strength, body weight) were used for validation. Results illustrate that untreated SOD1-G93A ALS dynamics cannot maintain homeostasis due to a mathematical oscillating instability as determined by eigenvalue analysis. The onset and magnitude of homeostatic instability corresponded to disease onset and progression. Oscillations were associated with high feedback gain due to hypervigilant regulation. Multiple combination treatments stabilized the SOD1-G93A ALS mouse dynamics to near-normal WT homeostasis. However, treatment timing and effect size were critical to stabilization corresponding to therapeutic success. The dynamics-based approach redefines therapeutic strategies by emphasizing the restoration of homeostasis through precisely timed and stabilizing combination therapies, presenting a promising framework for application to other multifactorial neurodegenerative diseases.

## 1. Introduction

Amyotrophic lateral sclerosis (ALS) is a complex multifactorial neurodegenerative disorder characterized by progressive loss of motor neurons. ALS exhibits vast heterogeneity in both its underlying etiology and its phenotypic manifestations [1]. The molecular etiology of ALS involves a dynamic interplay of several interdependent mechanisms that collectively drive disease progression. Apoptosis, the programmed cell death of motor neurons, is a key feature and often represents the final step in the cascade of cellular dysfunction. Disruptions in bioenergetics, including impaired mitochondrial function and alterations in energy metabolism, contribute to neuronal energy deficits. Aberrant chemistry, such as enzymatic dysfunction and mismanagement of metal ions such as zinc and copper, disrupts cellular homeostasis and amplifies toxicity. Excitotoxicity, resulting from excessive stimulation of glutamate receptors, leads to calcium overload and neuronal damage. Inflammation, driven by the activation of microglia and astrocytes, exacerbates the degenerative process through the release of pro-inflammatory cytokines. Oxidative stress, characterized by excessive production of reactive oxygen species and free radicals, further damages lipids, proteins, and DNA. Finally, abnormalities in proteomics, including protein misfolding and aggregation, contribute to the accumulation of toxic protein species, such as misfolded superoxide dismutase 1 (SOD1).

Over the past decades, traditional approaches to studying ALS have achieved limited success [2,3]. Currently, the main drugs for the treatment of ALS include riluzole, edaravone, and sodium phenylbutyrate–taurursodiol [3,4,5]. Riluzole may reduce glutamate levels, mitigating excitotoxicity, while edaravone, an antioxidant, may slow neuron damage. Sodium phenylbutyrate–taurursodiol, a combination drug, is believed to target mitochondrial dysfunction and endoplasmic reticulum stress. However, these treatments only modestly slow disease progression and provide limited improvements in quality of life [2,3].

High-copy superoxide dismutase 1 glycine 93 to alanine (SOD1-G93A) transgenic mice exhibit mutations associated with amyotrophic lateral sclerosis (ALS) [6]. However, a significant challenge of SOD1-G93A transgenic mouse experimental studies is the tendency to study isolated aspects of the disease rather than its multifactorial nature [7]. Mitchell and Lee [8], developed a preliminary model of the interactive physiology of the SOD1-G93A ALS mouse using categorical experimental data approximations, which identified an unstable system. Analyses suggested that failed homeostasis may be central to the underlying SOD1-G93A ALS disease etiology [8,9]. Specifically, the homeostatic instability hypothesis posits that SOD1-G93A ALS initiation and progression stems from an inability to maintain homeostasis following perturbation [9]. Motoneurons, due to their long axons and wide temporal dynamics, are particularly susceptible to instability [9]. In particular, clinical [10,11,12] and experimental associations [9,13,14] have suggested that ALS regulation may be hypervigilant, which means the pathological state attempts to over-correct to perturbation, leading to damaging oscillatory network behavior associated with pathological progression. Finally, electrophysiology experiments of SOD1-G93A ALS mouse motoneurons found increases in motoneuron gains [15,16] that indicate hypervigilant regulation.

Control of a biological system greatly relies on feedback control, where the system’s output is measured and used to regulate its input. Feedback can be either negative (counteract deviations) or positive (amplify changes). Gain determines the strength of the feedback signal relative to the deviation of the system’s output from the desired state. High gain can improve the system’s responsiveness but may lead to instability (oscillations or divergence). Low gain ensures stability but may result in slower corrections or insufficient control. Feedback systems with appropriate gain settings help maintain stability by ensuring that deviations are corrected without overshooting or oscillating excessively. In essence, feedback control allows biological systems to self-regulate, maintain homeostasis, and adapt to changes in the environment. Proper tuning of the physiological system’s gain is critical to achieving a balance between responsiveness and stability.

Feedback mechanisms are commonly modeled using first-order ordinary differential equations (ODEs), which describe how a system’s output evolves over time based on its current state and inputs [17]. This approach traditionally requires precise knowledge or experimental measurement of the gains for every feedback process in the system. In the case of ALS, many feedback gains have been measured, either directly or indirectly, using the widely studied transgenic SOD1-G93A mouse model. However, gaps remain where such measurements are unavailable.

This study employs an innovative, integrative framework to comprehensively investigate the regulatory dynamics of WT and SOD1-G93A ALS mouse models in silico. The research is guided by the hypothesis that ALS exhibits unstable regulatory dynamics, ultimately leading to homeostatic stability. By addressing critical gaps in the in silico modeling of SOD1-G93A ALS physiology, this study integrates dynamic meta-analysis [8] with genetic algorithm-based parameter optimization [18] to infer missing data.

Dynamic meta-analysis [8] synthesizes experimentally measured data from the literature into a physiology-driven ontology, which is then incorporated into first-order ordinary differential equations (ODEs) to model the time-dependent regulatory networks underlying ALS pathology. The genetic algorithm-based optimization refines undetermined parameter values, enabling the development of comprehensive in silico models that facilitate understanding and simulation of ALS progression.

The comprehensive in silico model facilitates the evaluation of therapeutic interventions on multifactorial regulatory dynamics. Previous ALS studies focusing on single therapeutic targets have often proven inadequate [14]. In contrast, combination treatments have demonstrated success in managing other complex diseases, including multimutation cancers, COVID-19, and HIV [19,20,21]. Addressing the intricate, multifactorial dynamics of ALS pathology likely requires precisely timed combination therapies that target multiple synergistic pathways to achieve meaningful and functional outcomes [8,22]. The sheer number of potential ALS combination treatments makes comprehensive in vivo exploration both cost-prohibitive and time-intensive, underscoring the critical value of a robust in silico model [23].

## 2. Results

**Figure 1:**
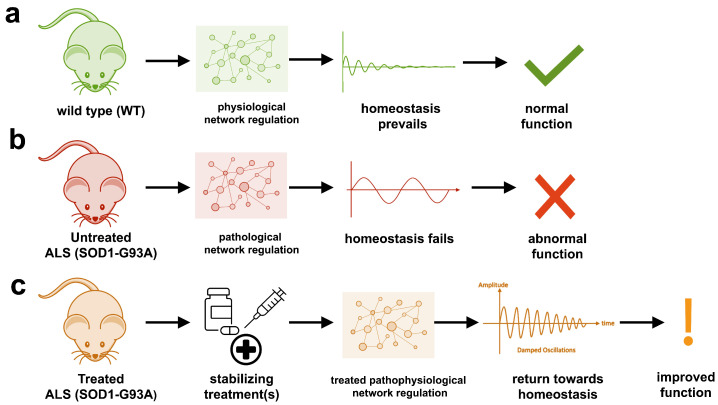
Study overview of the role of physiological network dynamics in health, disease onset and progression, and therapeutic response in SOD1-G93A ALS mice. Based on preliminary findings, we hypothesized that SOD1-G93A ALS mice exhibit unstable physiological regulation, contributing to disease onset and progression, whereas wild-type (WT) mice maintain normal, stable homeostatic regulation: (**a**) WT mice illustrate normal, homeostatic regulation. Overall system stability is maintained even when experiencing mild to moderate perturbations. (**b**) SOD1-G93A ALS mice illustrate unstable, oscillatory regulation. (**c**) In silico modeling of combination treatments for ALS, informed by experimental data from ALS mouse models, attempts to restore homeostasis to levels comparable to those observed in WT mice using treatments designed to stabilize the system dynamics.

The present study investigates the regulatory dynamics of the SOD1-G93A transgenic ALS mouse and its response to potential therapeutic interventions. Based on preliminary findings, we hypothesized that SOD1-G93A ALS mice exhibit unstable physiological regulation, contributing to disease onset and progression, whereas wild type (WT) mice maintain normal, stable homeostatic regulation. To test this hypothesis, two in silico models were developed: a WT mouse model to simulate normal homeostasis and a SOD1-G93A ALS model to explore the pathological instability associated with ALS and its response to potential treatments (see Figure 1).

**Figure 2:**
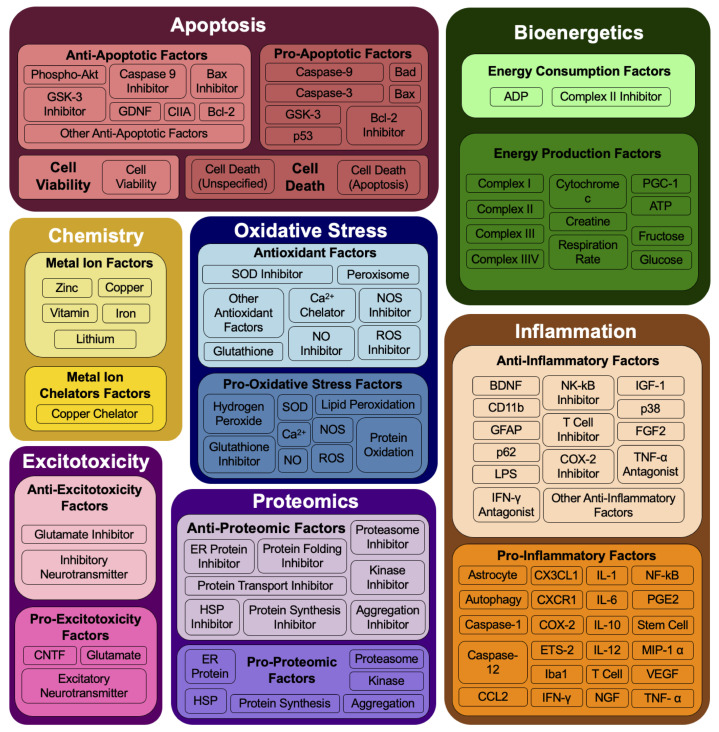
A functional ontology aggregates the multifactorial molecular mechanisms of the SOD1-G93A ALS mouse that impact disease progression. The ontology classifies the major interactive functional categories: apoptosis, bioenergetics, chemistry, excitotoxicity, inflammation, oxidative stress, and proteomics. Each category can be either positively or negatively regulated, resulting in 14 subcategories, collectively referred to as “factors” throughout the results. These subcategories are composed of 110 individual experimental measurements.

To construct the models, experimental data examining multifactorial molecular mechanisms of ALS in SOD1-G93A mice was curated from peer-reviewed literature. Curated data were divided into three datasets: model construction dataset (2148 data points; 119 articles); validation dataset (1477 data points; 180 articles); disease construction dataset (1850; 75 articles). An ontology (see Figure 2) was developed to classify the major interactive categories of molecular mechanisms: apoptosis, bioenergetics, chemistry, excitotoxicity, inflammation, oxidative stress, and proteomics. Each category can be either positively or negatively regulated, resulting in 14 subcategories, collectively referred to as “factors” throughout the results. These subcategories are composed of 110 individual experimental measurements (Figure 2).

**Figure 3:**
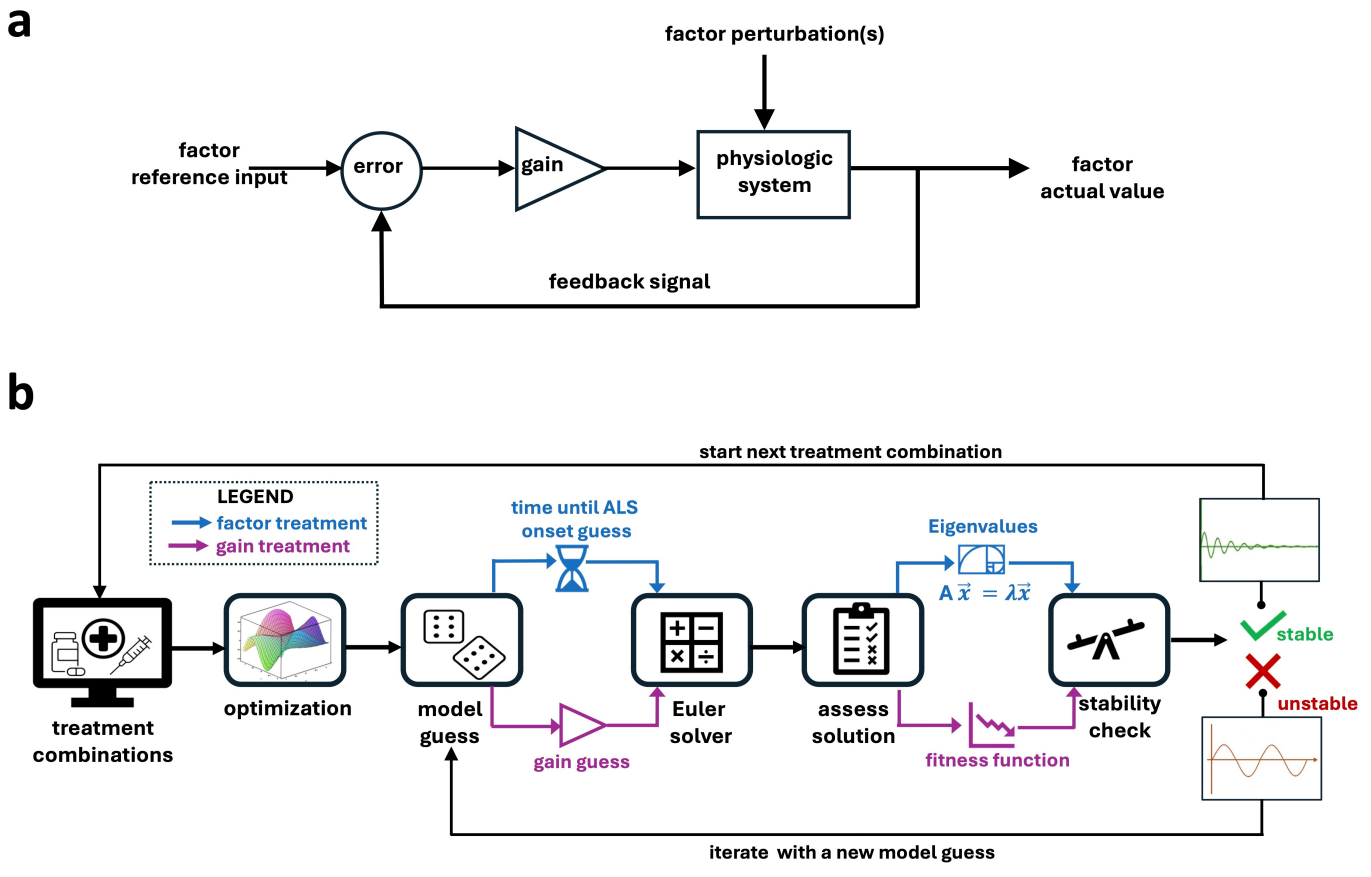
Modeling physiologic regulation in wild type (WT) and ALS SOD1-G93A ALS mice: (**a**) Schematic of a traditional first-order feedback system. First-order feedback systems regulate a process by continuously adjusting the output based on the difference between the reference input and the actual output. This difference, called the error signal, is amplified by a gain and processed by the controller to drive the system toward the desired reference input. (**b**) Schematic overview of the in silico model framework. The shared structural framework (depicted in black) underlies both factor-based treatments (shown in purple) and gain-based treatments (shown in blue). The objective of both approaches is to restore the system to a stable homeostatic state. The key distinction lies in the treatment target: factor-based treatments modify input factors, while gain-based treatments adjust system gains.

The primary first-order ODE structure for modeling the regulatory dynamics is: $\frac{d\vec{x}\left(t\right)}{dt}=[\mathrm{Gain}\;\mathrm{Matrix}]\cdot \vec{x}\left(t\right)$. The basic system control diagram is shown in Figure 3a. First-order feedback systems regulate a process by continuously adjusting the output based on the difference between the reference input and the actual output. This difference, called the error signal, is amplified by a gain and processed by the controller to drive the system toward the desired reference input. The feedback loop ensures dynamic adjustments to maintain stability and minimize the error signal over time.

Using first-order feedback, there are two primary strategies that can be used to influence the behavior of the physiological system: adjusting the gain values, [Gain Matrix] or modifying the input factors, $\vec{x}\left(t\right)$. Changing gain values directly affects the sensitivity and responsiveness of the system to deviations from equilibrium, essentially tuning how strongly the system reacts to feedback signals. This approach can enhance or dampen feedback from specific pathways. A higher gain creates a faster response to a deviation from the desired reference input; however, if the gain is too high, the controller continuously overshoots or undershoots the reference value, which creates destabilizing oscillations. Alternatively, modifying input factors alters the initial conditions or external stimuli driving the system, influencing the overall trajectory of the response. While gain adjustment fine-tunes internal regulatory mechanisms, input factor changes shift the baseline forces acting on the system. Together, these methods provide complementary avenues to modulate biological processes, offering flexible strategies to restore balance or achieve desired outcomes.

Most traditional real-world treatments aim to modify a physiological system by altering an input factor, with the resulting change in output measured as an effect size. In contrast, in silico modeling allows the exploration of both input factor modifications and gain adjustments to assess their effects on system output and stability over time. While direct gain modification is challenging in experimental settings, it can be modeled computationally to evaluate its impact on system dynamics and output. To comprehensively investigate the response of pathological dynamics to treatment, both factor-based and gain-based approaches are explored here in silico.

Figure 3b provides a schematic overview of the in silico model framework. The shared structural framework (depicted in black) underlies both factor-based treatments (shown in purple) and gain-based treatments (shown in blue). The objective of both approaches is to restore the system to a stable homeostatic state. The key distinction lies in the treatment target: factor-based treatments modify input factors, while gain-based treatments adjust system gains. Gain-based treatments are evaluated by calculating eigenvalues, using standard stability criteria that require all eigenvalues to have negative real parts for stability. Factor-based treatments, on the other hand, minimize the error of a fitness function to predict the time until ALS onset under the new conditions. Effect sizes are used to scale the degree of input factor or gain modulation required for system re-stabilization.

### 2.1. Untreated ALS Mice Have Unstable, Oscillatory Dynamics

**Figure 4:**
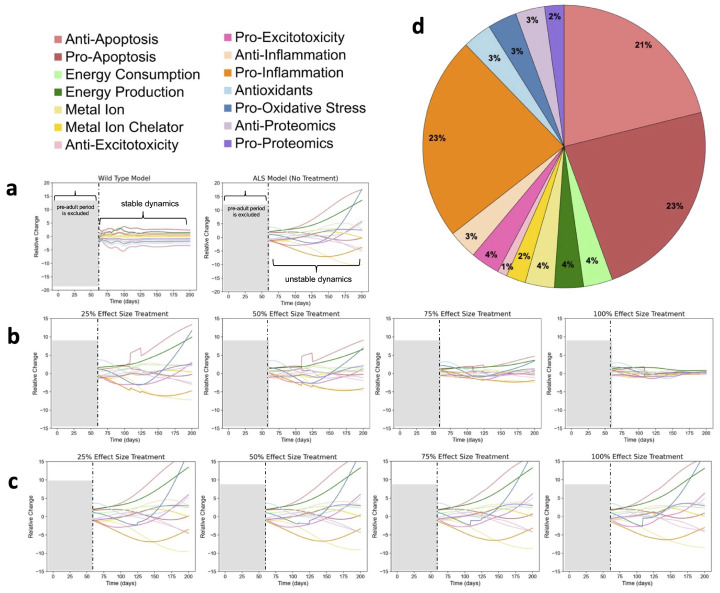
Examination of wild type (WT) and SOD1-G93A amyotrophic lateral sclerosis (ALS) mouse dynamics and the response of ALS system dynamics to factor-based combination treatments: (**a**) WT model (left) and untreated ALS model (right). The values of each of the 14 factors were plotted over the course of 200 days. Decreasing fluctuation of the factor values indicates increasing stability. The plots demonstrate stability for the WT model and instability for the ALS model in the absence of treatment. (**b**) Results for the most stable ALS factor-based treatment combination, which targeted anti-apoptosis, pro-apoptosis, and pro-inflammatory factors. Treatment effect sizes are shown left to right: 25%, 50%, 75%, and 100%. Higher effect sizes allow treatment to better mimic WT behavior. (**c**) Results for the least stable ALS factor-based treatment combination, which targeted metal ion chelator, anti-excitotoxicity, and pro-oxidative stress factors. Treatment effect sizes are shown left to right: 25%, 50%, 75%, and 100%. Increasing effect size has little effect on the stability of the treatment. (**d**) Treatment combinations were ranked based on stability. The frequency of occurrence of each factor in the top 30 most stable combinations is shown. Stability of each treatment combination was calculated based on similarity to WT model behavior.

Figure 4a,b illustrate the dynamics WT mouse model (shown on the left) and the untreated SOD1-G93A ALS mouse model (shown on the right). The results are color-coded for the functional categories shown in Figure 2. More data were missing for the early postnatal experimental time points. Regardless, during this time frame, it would be more difficult to disambiguate regulatory changes due to growth and development versus instability from inadequate homeostatic control. Thus, model results are most relevant for examining the dynamics of adult mice. Based on available data and optimization, the model is optimized to be the most accurate from approximately postnatal day 55 (early adulthood) through postnatal day 730 (end of WT mouse lifespan). However, in contrast, the average maximum lifespan for high-copy SOD1-G93A ALS mouse span is about 200 days. The graphs of Figure 4 focus on the most relevant time span of postnatal day 55 through postnatal day 200. As shown in Figure 4a, the 14 color-coded factors have mathematically stable dynamics. In contrast, the untreated SOD1-G93A ALS mouse has unstable dynamics as shown in Figure 4a (right). Notably, the visible trajectory towards system instability in the SOD1-G93A ALS mouse aligns with the average disease onset, which is typically around postnatal day 100 in high-copy SOD1-G93A mice [6]. These results corroborate the hypothesis that SOD1-G93A ALS disease onset and progression is a function of its underlying unstable multifactorial dynamics. Moreover, mathematical evaluation of system eigenvalues confirms that the SOD1-G93A ALS dynamics are indeed an oscillatory instability.

### 2.2. Stabilizing Treatments Highlight Potential Targets

As explained earlier in the Results Section, factor-based treatments modify input factors, while gain-based treatments adjust system gains. These methods provide two different ways to stabilize the regulatory dynamics of ALS to near-normal homeostasis.

#### 2.2.1. Factor-Based Stabilizing Treatments

Some combinations of factor treatments were able to stabilize the ALS mouse comparable to that of the WT mouse of Figure 4a. The combination treatments were ranked in descending order of stability. Analysis of the least stable treatment combination provides a baseline for how factor treatment combinations were performed. The most stable treatment combinations are shown in Figure 4b from left to right for 25%, 50%, 75%, and 100% treatment effect sizes. The least stable treatment combinations are shown in Figure 4c from left to right for 25%, 50%, 75%, and 100% treatment effect sizes. Based on fitness function values, the least stable treatment performed 1.04 times better than the untreated ALS model, while the most stable treatment performed 5.54 times better than the untreated ALS model. Thus, even the least stable treatment combination is still more stable than the untreated ALS model.

Combination treatments simultaneously target multiple synergistic pathways. The least effective treatment produced measurable improvement. Combination treatments become more effective as the effect size increases. Across multiple effect sizes, the same factors that play a role in stabilizing at lower effect sizes continue to play a role in stabilization at higher effect sizes. However, higher effect sizes lead to greater stability. Mathematically, there is a larger space to search for the optimum treatment parameters, as a higher effect size allows modulation of factors and gain values within a larger range. Biologically, the effect size simulates the strength or dosage of the treatment. A stronger treatment will produce a greater effect in treating ALS conditions.

Analysis of the frequency of occurrence for each of the 14 factors within the top 30 treatment combinations was performed (Figure 4d). Anti-apoptotic, pro-apoptotic, and pro-inflammatory factors were the most frequently involved in stable combinations, collectively accounting for 67% of all factors in stable combinations. In contrast, the least stable treatment combinations predominantly targeted metal ion chelation, anti-excitotoxicity, and pro-oxidative stress factors, which together comprised 51% of the factors in the 30 least stable combinations.

#### 2.2.2. Gain-Based Stabilizing Treatments

**Figure 5:**
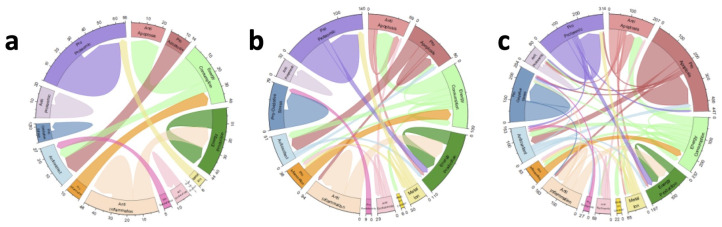
Gain treatment results. The chord diagrams illustrate the stable one-way and two-way gain treatments. The arcs indicate treatment combinations. For example, an arc starting at anti-apoptosis (light red) and going back to anti-apoptosis is the anti-apoptosis one-way treatment, while an arc starting at anti-apoptosis and going to metal ion chelator (dark yellow) is the anti-apoptosis-metal ion chelator two-way treatment. The thickness of the arc is the number of times that particular treatment stabilized. The colors match the chart illustrated in Figure 2. Briefly, clockwise from the top are: anti-apoptosis (light red), pro-apoptosis (dark red), energy consumption (light green), energy production (dark green), metal ion (light yellow), metal ion chelation (dark yellow), anti-apoptosis (light pink), pro-apoptosis (dark pink), anti-inflammation (light orange), pro-inflammation (dark orange), anti-oxidants (light blue), pro-oxidative stress (dark blue), anti-proteomic (light purple), pro-proteomic (dark purple): (**a**) Treatment combinations that stabilized for trials up to 5× effect size. (**b**) Treatment combinations that stabilized for trials up to 10× effect size. (**c**) Treatment combinations that stabilized for trials up to 15× effect size.

For gain treatment maximum effect sizes of 5×, 10×, and 15×, a maximum of 10, 24, and 50 combinations stabilized, respectively. The combinations that stabilized at smaller effect sizes continued to stabilize for larger maximum effect sizes, as shown in Figure 5. Combinations modulating gain values for anti-apoptosis, energy consumption, energy production, anti-inflammatory, and pro-proteomic factors stabilized most often, as shown in Table 1.

### 2.3. Time Series Clustering to Explore Patterns Among Re-Stabilizing Treatments

Post-simulation was necessary to examine the temporal patterns of stable treatment. This evaluation is necessary to (1) determine the optimal time of intervention for a given combination and (2) ensure that stability is maintained through the relevant biological lifespan.

K-means clustering was applied to the time series data to identify patterns associated with why certain treatment combinations stabilized the system, focusing on the time domain. Time series clustering, using dynamic time warping as the distance metric, resulted in consistent cluster assignments across the combinations. For the stable treatment combinations, factors such as energy consumption, metal ions, metal ion chelation, anti-excitotoxicity, anti-proteomic, and pro-proteomic were frequently clustered together. In contrast, anti-apoptotic, pro-apoptotic, pro-excitotoxicity, anti-inflammatory, pro-inflammatory, and antioxidant factors tended to form a separate cluster. Among the three identified clusters, two typically contained multiple factors, while the third cluster often consisted of a single factor or two unrelated factors.

Interestingly, factors with opposing roles were frequently grouped together within the same cluster. The most prominent example was the anti-proteomic and pro-proteomic pair, which clustered together in 47 out of the 50 stable treatment combinations. Similarly, other opposing pairs, such as metal ion and metal ion chelator, anti-excitotoxicity and pro-excitotoxicity, and anti-inflammatory and pro-inflammatory factors, were often grouped together. These findings suggest a potential regulatory interplay between opposing factors that contributes to system stability.

In some cases, the interplay between factors results in a synergistic effect, where the combined influence of opposing factors amplifies their overall impact. For example, an increase in anti-apoptotic factors alongside a decrease in pro-apoptotic factors enhances the regulatory influence of anti-apoptotic processes within the network. In other instances, the interaction involves a time-dependent component linked to correlated factors. Notably, anti-apoptotic and energy production factors show a strong positive Pearson correlation. However, these processes do not occur simultaneously; instead, one serially triggers the other. The time series data reveal a characteristic temporal shift between energetic pathways and apoptotic pathways. Consequently, the relative importance of opposing factors varies over time, depending on the specific stage of disease progression. This oscillatory temporal variability underscores the dynamic nature of the regulatory network in ALS pathology.

### 2.4. Eigenvalue Analysis of Stable Treatments

**Figure 6:**
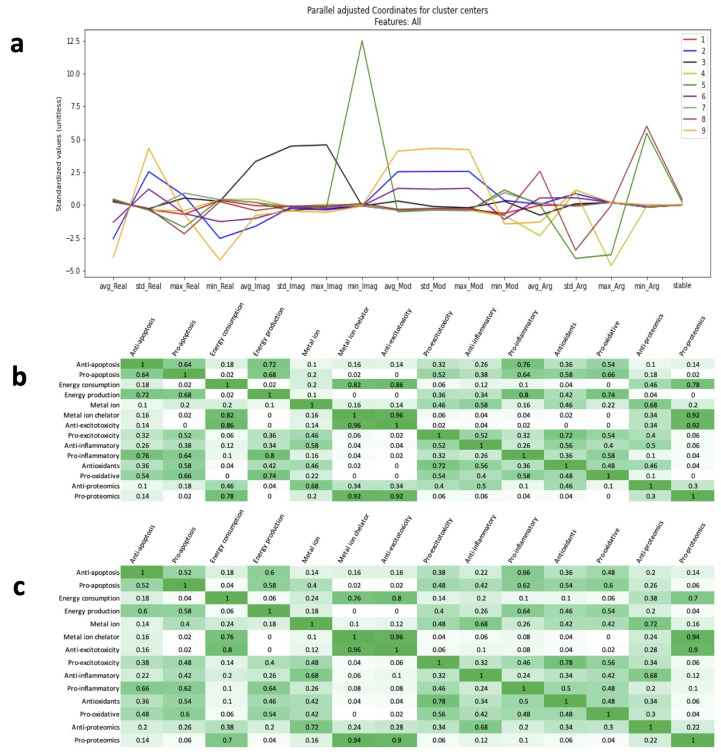
Eigenvalue clustering analysis: (**a**) Parallel centers plot for all complex characteristics with nine clusters. Stable treatments exist in clusters 5 and 8. By the definition of stability, maximum real component is relatively low for these clusters. The minium imaginary component feature shows that most stable treated systems will continue to oscillate. Cluster 5 and cluster 8 both sit around relatively low values for each standard deviation feature indicating that stable solutions tend to have tightly packed eigenvalues in the complex plane. (**b**) Frequency table for appearances in the same cluster for 50 stable treated systems by dominating magnitude at 100 days. (**c**) Frequency table for appearances in the same cluster for 50 stable treated systems by the dominating term by characteristic rate of decay. The groups of most probable factors in (**b**,**c**) are very similar with only a few discrepancies.

As expected, the clusters containing stable treatments exhibited the lowest maximum real eigenvalue, consistent with the definition of stability. These clusters also showed relatively large imaginary parts of eigenvalues and low standard deviations across all characteristics. The large imaginary parts indicate the absence of non-oscillatory stable solutions, while the low standard deviations suggest that the eigenvalues are tightly clustered in the complex plane (Figure 6a).

For the clustering based on magnitude at day 100 using K = 3 clusters, three distinct groups of factors were identified, each sharing similar dominant eigenterms from the analytic solution (Figure 6b). The first group included anti-apoptotic, pro-apoptotic, energy production, pro-inflammatory, antioxidants, and pro-oxidative stress factors. The second group consisted of energy consumption, metal ions, metal ion chelation, anti-excitotoxicity, anti-proteomics, and pro-proteomics. The final group included pro-excitotoxicity and anti-inflammatory factors.

For the clustering based on the rate of decay using K = 3 clusters, four groups of factors were identified, each sharing similar dominant terms Figure 6c. These groups were less distinct than those observed in the magnitude-based clustering. One group included pro-proteomics, energy consumption, metal ion chelation, and anti-excitotoxicity. The second group contained anti-apoptotic, pro-apoptotic, energy production, and pro-inflammatory factors. The third group comprised antioxidants, pro-oxidative stress, and pro-excitotoxicity. The fourth group included anti-inflammatory, metal ion, and anti-proteomics factors.

### 2.5. Spectrogram, Power Spectral Density, and Correlations

**Figure 7:**
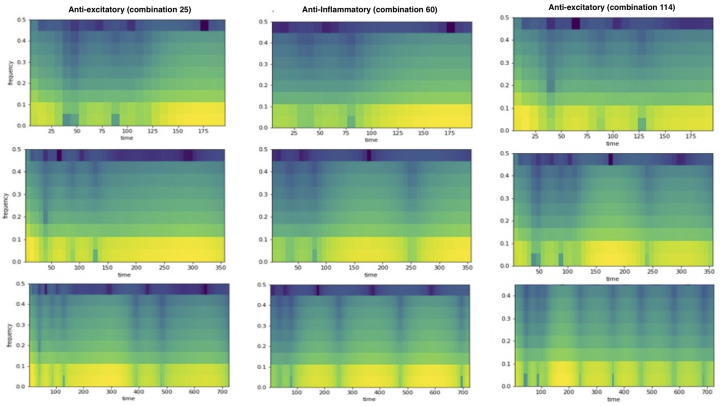
Spectrogram analyses. Spectrograms are shown for three factors with different lengths of days at different treatment combinations. The changes in the spectrogram patterns at different time lengths illustrate how stability can be affected by the timing of the applied treatment.

Figure 7 presents the spectrograms for three different factor pairs, emphasizing the lack of long-term stability after achieving momentary stability. The anti-excitatory signal appears stable after 140 days. However, when extended to 730 days, the signal begins to deviate from its stable position after 390 days. Similarly, the anti-inflammatory signal remains stable for 100 days, but upon extension to 365 days, it begins to deviate after 240 days. These observations underscore the importance of extending the stability analysis beyond 200 days.

**Figure 8:**
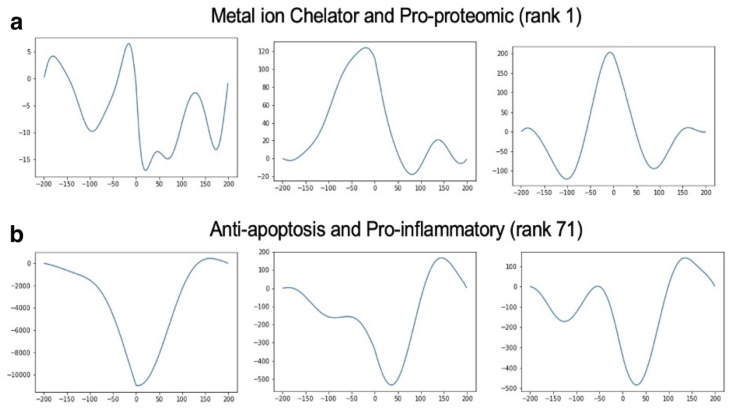
Cross-correlation between two factor pairs for different treatment combinations. Cross-correlation between two factor pairs that appear low and high in the aggregated ranking schema for three different treatment combinations. Metal ion chelator and pro-proteomic factors, which rank high in the aggregated ranking, have a distinctive peak at 0. On the other hand, anti-apoptosis and pro-inflammatory factors, which rank low in the aggregated ranking, have a distinctive trough at 0: (**a**) Metal ion chelator and pro-proteomic (rank 1). (**b**) Anti-apoptosis and pro-inflammatory (rank 71).

Periodic trends were observed in the time series data, particularly when the time period exceeded 200 days Figure 8. For many stable combinations, multiple factors exhibited the same amplitude and periodicity, but with phase shifts. This is evidenced by Pearson’s correlation values between pairs of factors. A highly negative correlation was found between anti-apoptosis and metal ion (−0.856), anti-apoptosis and pro-apoptosis (−0.752), and anti-excitotoxicity and pro-oxidative stress (−0.697). These correlations were calculated over a 200-day period. In contrast, a highly positive correlation was found between anti-apoptosis and energy production (0.752), metal ion chelator and pro-proteomics (0.736), and pro-apoptosis and metal ion (0.727). Extending the time series beyond 200 days yielded similar pairs of strongly negative or positive correlations. Pearson’s correlation results were consistent with those obtained using cross-correlation, dot product, and spectrogram analyses.

The biological relevance and accuracy of each combination of factors was assessed individually considering initial conditions, non-transient terms and frequency, and phase shift. Different physiological factors exhibit distinct time scales in their responses, leading to varying phase shifts. Notably, pro-apoptosis and anti-apoptosis factors are almost always out of phase, a relationship further supported by the above-noted negative Pearson correlation coefficient.

Spectrogram and factor pair correlations show that the factor signal values fluctuate from stable positions. An aggregate metric combining PSD, cross-correlation, and Pearson’s correlation was used to rank factor pairs by similarity. These factor pair correlation rankings are not identical for 200, 365, and 730 days (see Table 2). Only the top two most correlated factor pairs are the same across different simulation lengths, as shown in Table 2. The top two factor pairs, metal ion chelator with pro-proteomic and energy consumption with metal ion chelator, remained consistent across all simulation lengths. The metal ion chelator and pro-proteomic pair ranked in the top two for all three metrics. However, fluctuations in phase and frequency were observed even after achieving stability.

Notably, the spectrogram data demonstrate shifts in power distribution across different frequencies following temporary stabilization. These findings suggest that the relative importance of factors contributing to stability changes over time, emphasizing the need for time-specific therapeutic strategies. Consequently, the timing of treatment plays a critical role in determining which ALS molecular mechanisms should be targeted for optimal therapeutic outcomes.

## 3. Discussion

Results indicate that anti-apoptosis, pro-apoptosis, and pro-inflammatory factors are most often involved in stable combinations for factor treatments. The importance of apoptosis is corroborated by their appearance as predominant stabilizers in gain treatments. However, pro-proteomic, pro-oxidative, anti-inflammatory, and energy consumption factors play a much more important role in stable combinations for gain treatments. The best functional category to target varied over time. These findings illustrate the complex regulatory dynamics of SOD1-G93A ALS require carefully timed combination treatments to modulate the correct category at the most appropriate time to optimally stabilize the system using realistic treatment effect sizes.

### 3.1. Examination of Molecular Mechanisms Within Interactive ALS Dynamics

Neuroinflammation is commonly acknowledged to play a role in ALS. The role of the immune system in ALS can be divided into two phases—a neuroprotective phase and a neurotoxic phase [24,25,26,27]. In the neurotoxic phase, pro-inflammatory cytokines, such as various interleukins [28,29,30,31,32,33,34,35,36,37,38], INF- [28,29,38], and TNF- [28,39,40,41,42] are released, possibly leading to neurodegeneration and motoneuron degradation. However, the exact mechanism of action by which these inflammatory cytokines contribute to the progression of ALS is still unknown [43]. The results of the present study corroborate the important role of pro-inflammatory factors in ALS.

ALS regulatory network factors exhibit oscillatory behavior over time, often sharing the same frequency but differing in phase. For instance, simulation results demonstrate that anti-apoptosis and energy production factors oscillate with the same frequency and display similar waveforms in the time domain. This similarity explains their frequent clustering and their highly positive Pearson correlation. However, these factors differ in phase, with their oscillatory patterns being time-shifted relative to each other. Such time shifts reflect sequential activity, where one factor follows the other over time. These observed patterns in factor behavior are likely attributable to underlying time-dependent biological processes, highlighting the temporal dynamics of ALS pathology. These findings underscore previous work that emphasized the importance of feedback loops in physiological control dynamics [44], as well as pathology [22].

To understand the relationship between anti-apoptosis and energy production in a stable system, it is essential to examine the mechanisms underlying ALS pathology. In ALS, mitochondrial dysfunction plays a central role in initiating cell death. The mitochondria release apoptogenic proteins, such as cytochrome c [45,46,47], an energy production factor, which triggers downstream apoptotic pathways. This process involves the translocation of pro-apoptotic proteins [45,48], such as Bim and Bax, from the cytosol to the mitochondrial outer membrane. Their integration into the membrane increases its permeability, promoting pore formation and facilitating the release of additional pro-apoptotic proteins. The release of cytochrome c further activates pro-apoptotic caspases [49], which drive the degradation of intracellular proteins and DNA. This cascade ultimately leads to neuronal damage and the clinical manifestations of ALS. Consequently, in ALS systems, there is a direct positive relationship between energy production factors (e.g., cytochrome c) and pro-apoptosis factors (e.g., Bim, Bax, and caspases).

In contrast to the mechanisms observed in ALS pathology, the stable treatment simulations exhibit a direct positive relationship between energy production factors and anti-apoptosis factors. This relationship likely reflects a compensatory regulatory mechanism, wherein anti-apoptosis factors increase to counterbalance pro-apoptosis activity, thereby restoring homeostasis. Given the elevated release of cytochrome c in ALS pathology, stability in the system is achieved by upregulating anti-apoptosis factors such as caspase-9 inhibitor, caspase-3 inhibitor, Bax inhibitor, and Bcl-2 homolog BCL-XL. These factors act to prevent pore formation in the outer mitochondrial membrane, thereby limiting the release of apoptogenic proteins [45,48]. Notably, studies have demonstrated that overexpression of Bcl-2, an anti-apoptosis factor, can protect against motoneuron degeneration and apoptotic cell death [50], ultimately promoting stabilization of the ALS system. Consequently, in the most stable treatments, anti-apoptosis and energy production factors exhibit a highly positive Pearson correlation. However, as these processes do not occur simultaneously—one must trigger the other—the time series data reveal a characteristic time shift between these factors.

Furthermore, in the most stable treatments, anti-apoptosis and metal ion factors exhibit a highly negative Pearson correlation. Mutations in the copper and zinc superoxide dismutase 1 (SOD1) gene are known to contribute to ALS pathology [51]. The deficiency of copper and zinc ions is known to exacerbate the SOD1-G93A ALS pathology. Namely, the dissociation of copper and zinc ions from SOD1 leads to the aggregation of misfolded SOD1 proteins, a hallmark of ALS [52,53]. The observed negative correlation between metal ion factors and anti-apoptosis factors in stable treatments may reflect the system’s compensatory response, where increased anti-apoptosis activity is required to mitigate the pathological effects of disrupted metal ion homeostasis and ensure system stability.

### 3.2. Model and Results Applications: Reconceptualizing ALS Therapeutic Strategies

The primary objective of this study was to determine whether dynamic mathematical instability resulting from homeostatic dysregulation is present in the SOD1-G93A ALS transgenic mouse model and to evaluate its implications for therapeutic strategies. The results confirm the presence of dynamic instability, characterized by oscillatory behavior due to hypervigilant regulation induced by excessive feedback gains. This destabilization intensified near disease onset (approximately 100 days of age) and worsened with progression. Treatment simulations demonstrated that multiple combinations of interventions could stabilize the model, but effective strategies varied in molecular mechanisms, degree of modulation, and timing. These findings highlight the need for a paradigm shift in ALS treatment strategies.

Current experimental and clinical ALS therapies often target a single physiological mechanism, but the oscillatory nature of ALS implies that treatment effects vary across the disease timeline. For example, riluzole, which mitigates excitotoxicity, is more effective during the early stages of ALS but less impactful later [54]. Consistent with previous findings [14], this study showed that excitotoxicity is more pronounced near disease onset in SOD1-G93A mice. These results underscore the importance of developing “treatment windows” tailored to specific disease stages. Although the narrow standard deviation of onset in SOD1-G93A mice allows precise testing, the rates of onset and progression of human ALS vary widely due to genetic, environmental, and demographic factors. Therefore, the timing of therapy in humans should align with the relative stages of disease progression, using methodologies such as event-based modeling [55].

This study used a first-order feedback ODE dynamic meta-analysis to model high-copy SOD1-G93A ALS mice, which emulate familial SOD1-G93A mutations in humans [6]. The specific treatments for SOD1-G93A ALS may not generalize to other ALS subtypes (e.g., c9orf72 or TDP-FUS mutations) [56]. However, it is expected that the framework and approach to prioritize system stability will generalize to other mutations. In particular, while the initial perturbation in SOD1-G93A mice is known, targeting the associated metal ions regulatory mechanism alone failed to stop disease progression in silico. Stabilization required carefully timed and combined treatments, emphasizing that addressing downstream destabilizing dynamics is more critical than only targeting the initial perturbation.

The model also revealed that perturbations that trigger regulatory cascades can originate from diverse molecular mechanisms, suggesting multiple pathways to the ALS phenotype. Unlike wild-type regulation, which maintains stability, the hypervigilant regulation of SOD1-G93A ALS systems overcorrect perturbations, which causes oscillatory instability. Motoneurons, particularly vulnerable due to their length and regulatory time scales, are disproportionately affected [9]. The excessive feedback gains identified here aligned with direct measurements in ALS motoneurons [15,16].

In conclusion, this study highlights the need to reconceptualize therapeutic strategies for multifactorial diseases like ALS. Rather than prioritizing the identification of initial perturbations, treatments should focus on stabilizing dysregulated system dynamics, as evidenced by eigenvalue analysis. This dynamics-based approach shifts the emphasis from isolated molecular targets to restoring homeostasis through carefully timed combination therapies, offering a promising avenue for managing complex diseases.

The framework developed here, validated with experimental SOD1-G93A mouse data from peer-reviewed studies, provides a foundation for extending dynamic stability analyses to other ALS models or multifactorial clinical data. By prioritizing system stabilization, this strategy has the potential to transform ALS therapy and improve outcomes in other complex diseases.

### 3.3. Limitations and Future Work

The dynamic meta-analysis model framework employs first-principles-based first-order feedback. Real experimental SOD1-G93A ALS mouse data were used where possible, but optimization was required to infer missing data. The level of confidence in the data sources is listed in Table A1. Given the required simplifying assumptions, first-order feedback models may overlook some of the rich complexity of stability and resilience in biological systems. Many biological systems, including motoneurons [57], have multiple stable or unstable states (e.g., bistability), which first-order feedback models may fail to fully capture. Notably, the present work found multiple stable solutions for treating the ALS system. Real systems may exhibit hysteresis (e.g. dependence on the history of changes), a phenomenon not fully accounted for in simplistic stability assumptions. Finally, first-order feedback assumptions may not fully capture emergent properties that result from multilevel interactions not fully captured by the model ontology.

Future work should focus on matching specific pharmaceutics to the broader categories defined here. Such pharmaceutics would act as modulatory agents to stabilize the SOD1-G93A ALS multifactorial dynamics to normal or near-normal homeostasis. Subsequently, such prioritized combination therapies will need to be experimentally evaluated in SOD1-G93A transgenic mice.

## 4. Materials and Methods

In silico model WT and SOD1-G93A mice were developed using curated published literature and a novel method that integrated dynamic meta-analysis with genetic algorithm-based parameter optimization. Data curation, model construction, and analysis are detailed below.

### 4.1. Data Curation

Data curation protocols were based on prior works that built a database of ALS SOD1-G93A extra literature data [9,13,14,58]. Briefly, inclusion criteria were developed to compile a specific and accurate dataset for this study. A PubMed search of peer-reviewed articles was conducted using specified keywords in the title or abstract (“Amyotrophic Lateral Sclerosis” or “ALS” and “mouse” or “G93A”). This yielded approximately 3400 articles. Quantifiable data from relevant articles included in vitro and in vivo studies. Quantitative data were extracted using a previously published annotation pipeline and quality control procedures [14,58]. Extracted experimental groups included SOD1-G93A transgenic mouse control group, a SOD1-G93A transgenic mouse treatment group, a wild-type (WT) group, and a WT treatment group. Annotators extracted and curated tabular data and quantitative results from figures (bar charts, scatter plots, etc.) into a relational database [14,58]. Figure data were extracted from graphs using an automated figure data extraction tool [58]. Quality control analysts assessed the accuracy of the extracted data and evaluated their confidence in the alignment of the extracted data with their assigned functional ontology (Table A1). Table A1 also lists the relationships, sign, confidence level, and data source literature citation(s). The data were collected for three subsets, including model construction, model validation, and disease stage construction.

### 4.2. Functional Ontology to Aggregate SOD1-G93A Mechanisms

Definitions for the functional ontology shown in Figure 2 were adapted from prior foundational works performing large-scale meta-analyses on SOD1-G93A transgenic mouse data [8,9,14]. The ontology aggregated multiscalar, multifactorial molecular mechanisms and pathways into a function-based ontology used as model input:

- Apoptosis: relating to or leading to cell death.
- Bioenergetics: relating to cellular processes that harvest and transform energy from cellular respiration and other metabolic processes and result in the production and utilization of energy.
- Chemistry: relating to enzymatic and metal mishandling.
- Excitotoxicity: relating to cell damage caused by the excitatory neurotransmitter.
- Inflammation: relating to the immune system.
- Oxidative stress: relating to reactive oxygen species and production of free radicals.
- Proteomics: relating to protein folding, aggregation, or degradation.

Although not part of the above ontology, systemic function metrics were included as part of a separate disease construction dataset. Experimental measures of neuromuscular disease progression over time included mouse rotarod performance, latency to fall, and body weight [6].

### 4.3. Partitioning of Data Sets

The curated quantifiable experimental data from the literature were assigned to one of three datasets:

- Model construction dataset—The model construction subset exclusively included data that contained control and treated data for high copy SOD1-G93A and WT mice. The control data were obtained from experiments that did not administer treatments or processes that could change the measured outputs. The treated data included experiments that provided effects of different perturbations. The specified criteria resulted in a final model construction data pool of 2148 data points from 119 articles.
- Validation dataset—The validation dataset was an external dataset not used in model construction that contained data points that could be used to validate model output. The dataset contained peer-reviewed articles that did not meet the inclusion criteria for the model construction dataset but had quantifiable output data relevant to the evaluation of model performance. The validation dataset had 1477 data points from 180 articles.
- Disease construction dataset—The disease stage construction data subset included figures from experiments that contained systematically measured response data (i.e., rotarod, latency to fall, and body weight). This criterion provided peer-reviewed articles that included the systemic deterioration of measured mice subjects and provided a visual representation of disease progression. The disease construction dataset yielded a total of 1850 data points from 75 articles.

### 4.4. Model Construction

The in silico mouse model is composed of a system of ordinary differential equations (ODEs) inspired by dynamic meta-analysis [8]. The system of ODEs describes the change in each factor over time relative to day 1, which is the birth of the mouse. However, the results from day 1 to 54 were excluded due to insufficient data to differentiate expected perturbations from growth and development from normal regulatory responses. Interactions among these factors create a matrix of coefficients, called the gain matrix. Coefficients calculated through extracted raw data formalized the foundation of the computational model with Equation (1). The function $\vec{x}\left(t\right)$ is a vector that represents the relative change in each factor over time. The gain matrix, [Gain Matrix], contains the gains for all interactions. The ordinary differential equation system is solved numerically with Euler’s method (Figure 3b).

(1) $$\frac{d\vec{x}\left(t\right)}{dt}=[\mathrm{Gain}\;\mathrm{Matrix}]\cdot \vec{x}\left(t\right)$$

To capture daily gain or rate of change, the gain was calculated from the raw ratio of treated and control values using Equation (2).

(2) $$\mathrm{Gain}=3*\mathrm{Days}\sqrt{\frac{\mathrm{Treated}}{\mathrm{Control}}}-1$$

The time scale of transition is proportional to $\frac{1}{\lambda_{1}-\lambda_{2}}$, which was determined to range between 1000 and 10,000 days. Importantly, for t>0, the non-transient terms consistently exceed the transient terms, ensuring that the dominant frequencies dictate the long-term physiological dynamics.

Equation (2) used data from peer-reviewed experimental SOD1-G93A ALS studies to calculate the values of the gain matrices. However, some values were missing due to the lack of published experimental data for some relationships. Using known WT and ALS behaviors, these missing values can be inferred using optimization with a genetic algorithm, which has been successfully applied to many problems of high complexity [17,18].

Since a WT mouse is expected to maintain homeostasis over time, the values of the missing coefficients were optimized with a genetic algorithm to obtain solutions that stabilize the physiological system. A genetic algorithm is an optimization technique inspired by natural evolution and has been successfully applied to many problems of high complexity [18]. The genetic algorithm search was applied to the WT gain matrix to minimize a cost function given by mean squared error (MSE) (Equation (3)).

(3) $$MSE=\frac{1}{n}\sum_{i=1}^{n}{\left(x_{i}^{\prime }\left(200\right)-{\hat{x}}_{i}^{\prime }\left(200\right)\right)}^{2}\;\mathrm{with}\;{\hat{x}}_{i}^{\prime }\left(200\right)=0$$

### 4.5. Combination Treatment Simulations

Two methods were employed to test potential combination treatments in the ALS mouse. The factor-based method modulates values of the 14 factors at specific time points while the gain-based method modulates the gain values of the gain matrix as shown in Figure 3b.

### 4.6. Factor Treatments

Different combinations of factors in the ALS model were modulated to assess the ability of the system to achieve stability. These treatments targeted combinations of three of 14 factors at a time, resulting in a total of 364 distinct treatments. Additionally, five different treatment effect sizes were tested for each combination. To simulate disease progression, modulations that mimic therapeutic treatments were applied at time points corresponding to the presymptomatic, symptomatic, and post-symptomatic stages of ALS. These time points were systematically determined to be days 1, 109, and 125, respectively. Euler’s method was applied at these time points using the modulated factors to evaluate the stabilization of the system over a 200-day period.

To provide the best set of modulated factors, a differential evolution (DE) algorithm was implemented to search for the best-optimized factors. The DE algorithm utilized Equation (4), where $\hat{y}$ is the optimized treatment values at time points from days 140 to 200, and y is the corresponding WT values at the same respective time points. This was used to determine mathematical stability for the ALS system at the different disease progression time points.

(4) $$fitness=1/n\sum_{i=1}^{n}\log(cosh\left(y-\hat{y}\right)$$

### 4.7. Gain Treatments

Different combinations of SOD1-G93A ALS mouse model gain values were modulated to simulate system stability. The selected combinations included (1) a 1-way treatment, defined by a gain value along the diagonal of the gain matrix, and (2) a 2-way treatment, consisting of all other values in the matrix excluding the diagonals, resulting in a combined 3-way treatment.

### 4.8. Post-Simulation Data Analysis

The results of the treatment simulations were analyzed using various mathematical, machine learning, and pattern analysis techniques. The goal was to elucidate the interactions between different factors, physiological scales, and temporal scales to help predict disease progression and varying phenotypes. All data analysis was performed in Python 3.11.

#### 4.8.1. Stability Ranking of Factor Treatments

All 364 factor treatments were ranked by how well a treatment could stabilize the ALS model. The measure of how well a treatment would stabilize the ALS model was given by the fitness function value, shown in Equation (3). For each effect size, the 364 treatments were ranked by their fitness function value. The aggregated ranking of each treatment was determined by the sum of ranks across all 5 effect size values Table 2.

#### 4.8.2. Time Series Analysis

K-means clustering was performed on time series data to determine if there were time-based patterns. For each of the stable combinations, the optimized treatment gain values were inserted into the gain matrix. Using Euler’s method, the factor values were calculated for a period of 200, 365, 730, 1825, and 3650 days. Plotting the values of the 14 factors against time created sinusoidal plots. Clustering was performed in Python SciKitLearn using the tslearn.clustering function with 3 clusters and dynamic time warping (DTW) as the distance metric [59].

#### 4.8.3. Eigenvalue Analysis

Gain-treated systems have different sets of complex eigenvalues. The real part of the eigenvalues determines the stability of the system and was minimized by differential evolution optimization. Complex numbers are characterized by any two of the following: real part, imaginary part, complex modulus, and complex argument. Using K-means clustering, each characteristic was first clustered separately with features of minimum, maximum, mean, and standard deviation. Then, all four sets of features were combined to cluster the treatments on all characteristics simultaneously as shown in Figure 6.

Equation (1) also has an analytic solution, which is shown in Equation (4). The frequency of oscillation for each term in the sum is determined by Im(λ). The magnitude of each term compared with the others determines with which frequency each factor will oscillate. The magnitude is a function of time with some terms dying quickly and others dying much more slowly. The average lifetime for an ALS mouse is 200 days. Thus, comparing the magnitudes at the temporal order of 100 days provides an indication of which terms dominate during the onset of ALS.

(5) $$\vec{x}\left(t\right)=\sum_{i}c_{0,i}e^{\lambda_{i}t}{\vec{v}}_{i}$$

To account for an arbitrary initial condition, Equation (5) shows how the general magnitude of the ith term of the jth factor was calculated without the $c_{0,i}$

(6) |x→j(100)|i≈eRe(λi)·100vi,j

The terms within five percent of the largest magnitude for each factor were identified for each stable treatment and marked as the dominant terms. The Boolean value of whether a term was identified as dominant or not became a set of features for K-means clustering. For slowly dying terms, it is as follows:

(7) |Re(λi)t|≫(Re(λi)t)2

In a stable system, the analytic solution can be approximated. For stable systems, the slowest terms satisfy this condition. The characteristic rate of decay for these terms is then

(8) ∥Re(λi)|vi,j

This again ignores information about the initial condition for generality. Just as before, this value is calculated for each term for each factor. The smallest characteristic rate of decay and any terms within five percent were marked as the dominant terms. Clustering was again performed on features corresponding to Boolean values to determine if the term was “dominating”.

(9) x→(t)≈∑ic0,i1−|Re(λi)|tv→i

For both methods, the clustering algorithm identified factors that tended to oscillate together. Even in stable homeostasis, there are some fluctuations; knowing what factors fluctuate together provides additional molecular mechanism insight. The clustering was repeated separately for all 50 stable treatment combinations, and the frequency of factors clustered together was calculated.

#### 4.8.4. Spectrogram, Power Spectral Density, and Correlations

Three metrics, power spectral density, cross-correlation at zero phase difference, and Pearson’s correlation, were used to evaluate the similarity between the signals in different ways. These metrics were combined to obtain an aggregated ranking. Instead of combining the normalized raw metric values, the individual rankings were aggregated to obtain the final position of each factor pair.

Spectrograms generated using Short-Time Fourier Transform (STFT) were used to quantify and visualize the change in a signal’s local frequency content over time. The signal obtained for each factor was split into segments with a length of 16 days. The amount of overlap between subsequent sections was 8 days. The spectrogram shows the change in power across different frequencies over time and can be used to observe the stability of signals over time. It highlights potential fluctuations after obtaining stability. During ranking, the overlap between segments was minimized to 2 days. The spectrogram was normalized by frequency to obtain the power spectral density (PSD). The difference in PSD for different factors at different time points and across the frequency range was calculated. These differences were summed to obtain an overall discrepancy in PSD. These values were used to rank the factors in pairs.

Cross-correlation was used to observe the change in similarity between factor pairs across different phases. For similar factors, a peak was observed at zero. The cross-correlation value at zero corresponds to the dot product between the factor pairs and was used to rank the pairs of factors. This represents the second of the 3 metrics used for ranking aggregation and is the simplest similarity metric used in the ranking scheme.

Pearson’s correlation was calculated between pairs of factors for different time periods. With 14 factors, this resulted in 105 unique pairs of factors, including factors paired with themselves. For each of the stable combinations, the optimized treatment gain values were inserted into the gain matrix. Using Euler’s method, the factor values were calculated for a period of 200, 365, 730, 1825, and 3650 days. While the average lifespan of a WT mouse is estimated at about 1 year, extended time periods were plotted to help understand and visualize patterns over time.

## 5. Conclusions

This study hypothesized that system dynamics could predict SOD1-G93A ALS mouse disease onset, progression, and response to therapeutic modulation. To comprehensively assess this hypothesis, computational models of the physiology dynamics of wild-type (WT) and SOD1-G93A ALS transgenic mice were built using the first-principles-based first-order ODE feedback framework of dynamic meta-analysis [8] with parameter optimization. Data used to build the models were extracted from peer-reviewed SOD1-G93A transgenic ALS mouse publications. Key results included the following:

- SOD1-G93A ALS transgenic mice have an unstable multifactorial regulatory network that cannot maintain normal homeostasis. The pathology dynamics, including the onset of instability and magnitude of instability, correspond to disease progression.
- SOD1-G93A ALS transgenic mice have unstable regulatory dynamics primarily due to hypervigilant regulation (e.g., too high gains) that induce oscillatory, homeostatic instability.
- Mathematical stability based on the regulatory system’s eigenvalues was used as a criterion to determine whether a treatment is likely to be successful. Successful combination treatments stabilized the underlying network physiology of SOD1-G93A ALS transgenic mice to normal or near-normal homeostasis, similar to WT mice.
- The timing and effect size of modulatory treatment combinations is critical for optimizing therapeutic success (stabilization). The top two factor pairs, metal ion chelator with pro-proteomic and energy consumption with metal ion chelator, remained consistent across all simulation lengths. However, fluctuations in phase and frequency were observed even after achieving stability.

The underlying computational framework and the concept of dynamic regulatory physiological stabilization as a cornerstone for the therapeutic success of multifactorial diseases may also be applicable to other conditions, including Alzheimer’s disease, Parkinson’s disease, and multiple sclerosis.

## Figures and Tables

**Table 1:** The top 3 one-way treatments and two-way treatments that stabilized most often for each effect size. For example, for 5× effect size, the one-way treatment that stabilized most often was pro-proteomic, while the two-way treatment that stabilized most often was energy consumption and anti-apoptosis.

5× Effect Size	10× Effect Size	15× Effect Size
Pro-proteomic	Pro-proteomic	Pro-apoptosis
Anti-inflammatory	Pro-oxidative	Pro-proteomic
Energy production	Anti-inflammatory	Pro-oxidative
**Two-Way Treatment**		
Energy Consumption and Anti-Apoptosis	Energy Consumption and Anti-Apoptosis	Anti-Inflammatory and Energy Production
Anti-Inflammatory and Energy Production	Anti-Inflammatory and Energy Production	Energy Consumption and Anti-Apoptosis
Pro-Apoptosis and Antioxidant	Pro-Apoptosis and Antioxidant	Pro-Apoptosis and Antioxidant

**Table 2:** Aggregated ranking of factor pairs across all stable treatment combinations. Different factor pairs have different rank values over the course of a total of 730 days. The top two factor pairs, metal ion chelator and pro-proteomic and energy consumption and metal ion chelator, are consistent across all simulation lengths. Metal ion chelator and pro-proteomic factors appear in the top two ranks for all three metrics. The change in ranking across different treatment lengths shows that there are fluctuations in phases or frequency even after obtaining stability.

Treatment Combination	Rank @ 200 Days	Rank @ 365 Days	Rank @ 730 Days
Metal Ion Chelator + Pro-Proteomics	1	1	1
Energy Consumption + Metal Ion Chelator	2	2	2
Energy Consumption + Pro-Proteomic	3	5	3
Anti-Apoptosis + Energy Production	4	8	-
Pro-Excitotoxicity + Pro-Inflammatory	5	4	7
Anti-Excitatory + Anti-Proteomic	6	6	4
Energy Production + Pro-Proteomic	7	-	-
Metal Ion Chelator + Anti-Proteomic	8	7	5
Energy Consumption + Anti-Excitatory	9	3	6

## Data Availability

Additional supporting data may be accessed from the corresponding author or at https://github.com/pathology-dynamics accessed on 27 December 2024.

## Appendix A

### Appendix A.1

**Table A1:** Citation of data sources for construction of the WT and SOD1-G93A computational models. Treatment indicates the input category being modulated via in silico therapy. Measure indicates the output functional category. The sign denotes the directionality of the relationship as either positive (+) or negative (−). The confidence level reflects the degree of reliability assigned based on the quality and/or relevance of the experimental data in the data source citation(s). Confidence scale: 1 = no source citation available (listed as N/A under source citations; 2 = minimal relevant data available; 3 = adequate data available; 4 = good relevant data from multiple sources; 5 = excellent relevant data available from multiple sources.

Treatment	Measure	Sign +/−	Confidence	Source Citation
Anti-Apoptosis	Energy Consumption	+	5	[60,61]
Anti-Apoptosis	Ion	−	1	N/A
Anti-Apoptosis	Metal Ion Chelator	+	1	N/A
Anti-Apoptosis	Anti-Excitotoxicity	−	5	[62,63]
Anti-Apoptosis	Antioxidants	+	4	[64,65,66]
Anti-Apoptosis	Anti-Proteomic	−	2	[67]
Pro-Apoptosis	Energy Consumption	−	3	[60,68]
Pro-Apoptosis	Metal ion	+	4	[69,70]
Pro-Apoptosis	Metal Ion Chelator	−	1	N/A
Pro-Apoptosis	Anti-Excitotoxicity	−	2	[71]
Pro-Apoptosis	Anti-Inflammatory	+	2	[72]
Pro-Apoptosis	Pro-Inflammatory	−	4	[73,74]
Pro-Apoptosis	Antioxidants	+	3	[75]
Pro-Apoptosis	Pro-Oxidative	−	3	[76,77]
Pro-Apoptosis	Anti-Proteomic	−	4	[78,79]
Energy Consumption	Anti-Apoptosis	+	4	[80,81]
Energy Consumption	Pro-Apoptosis	−	4	[82,83]
Energy Consumption	Energy Consumption	−	2	[84]
Energy Consumption	Energy Production	+	4	[85,86]
Energy Consumption	Metal Ion	+	4	[87,88]
Energy Consumption	Metal Ion Chelator	−	2	[89]
Energy Consumption	Anti-Excitotoxicity	+	4	[90,91]
Energy Consumption	Pro-Excitotoxicity	−	3	[92,93]
Energy Consumption	Anti-Inflammatory	+	4	[94,95]
Energy Consumption	Pro-Inflammatory	+	4	[96,97]
Energy Consumption	Pro-Oxidative	+	4	[98,99]
Energy Consumption	Anti-Proteomic	−	2	[100]
Energy Consumption	Pro-Proteomic	−	4	[101,102]
Energy Production	Metal Ion	+	3	[103]
Energy Production	Metal Ion Chelator	−	1	N/A
Energy Production	Anti-Excitotoxicity	−	4	[104,105]
Energy Production	Anti-Inflammatory	−	2	[106]
Energy Production	Antioxidants	+	3	[107]
Energy Production	Anti-Proteomic	−	1	N/A
Energy Production	Pro-Proteomic	+	3	[108]
Metal Ion	Pro-Apoptosis	+	4	[109,110]
Metal Ion	Energy Consumption	−	4	[111,112]
Metal Ion	Energy Production	−	4	[113,114]
Metal Ion	Metal Ion	+	1	N/A
Metal Ion	Metal Ion Chelator	−	1	N/A
Metal Ion	Anti-Excitotoxicity	−	4	[115,116]
Metal Ion	Anti-Inflammatory	−	4	[117,118]
Metal Ion	Antioxidants	−	5	[119,120]
Metal Ion	Anti-Proteomic	−	4	[121,122]
Metal Ion	Pro-Proteomic	−	5	[123,124]
Metal Ion Chelator	Pro-Apoptosis	+	4	[125,126]
Metal Ion Chelator	Energy Consumption	+	4	[127,128]
Metal Ion Chelator	Metal Ion Chelator	+	NA	[129,130]
Metal Ion Chelator	Anti-Excitotoxicity	−	4	[131]
Metal Ion Chelator	Pro-Excitotoxicity	−	3	[132,133]
Metal Ion Chelator	Anti-Inflammatory	+	4	[134]
Metal Ion Chelator	Antioxidants	+	2	[135]
Metal Ion Chelator	Anti-Proteomic	+	3	[136]
Metal Ion Chelator	Pro-Proteomic	−	3	[118]
Anti-Excitotoxicity	Pro-Apoptosis	−	4	[118,137]
Anti-Excitotoxicity	Energy Consumption	+	4	[138,139]
Anti-Excitotoxicity	Energy Production	+	4	[81,140]
Anti-Excitotoxicity	Metal Ion	+	1	N/A
Anti-Excitotoxicity	Metal Ion Chelator	−	1	N/A
Anti-Excitotoxicity	Anti-Excitotoxicity	+	1	N/A
Anti-Excitotoxicity	Anti-Inflammatory	−	4	[141,142]
Anti-Excitotoxicity	Pro-Inflammatory	−	4	[143,144]
Anti-Excitotoxicity	Antioxidants	+	2	[145]
Anti-Excitotoxicity	Pro-Oxidative	+	2	[146]
Anti-Excitotoxicity	Anti-Proteomic	−	1	N/A
Anti-Excitotoxicity	Pro-Proteomic	+	4	[147,148]
Pro-Excitotoxicity	Anti-Apoptosis	−	4	[149,150]
Pro-Excitotoxicity	Energy Consumption	−	1	N/A
Pro-Excitotoxicity	Energy Production	+	4	[151,152]
Pro-Excitotoxicity	Metal Ion	+	2	[153]
Pro-Excitotoxicity	Metal Ion Chelator	−	1	N/A
Pro-Excitotoxicity	Anti-Excitotoxicity	+	4	[154,155]
Pro-Excitotoxicity	Anti-Inflammatory	+	3	[156]
Pro-Excitotoxicity	Pro-Inflammatory	+	5	[157,158]
Pro-Excitotoxicity	Antioxidants	+	4	[159,160]
Pro-Excitotoxicity	Anti-Proteomic	+	4	[161,162]
Anti-Inflammatory	Energy Production	+	3	[163]
Anti-Inflammatory	Metal Ion	−	3	[164]
Anti-Inflammatory	Metal Ion Chelator	+	1	N/A
Anti-Inflammatory	Anti-Excitotoxicity	+	2	[165]
Anti-Inflammatory	Antioxidants	+	4	[166,167]
Anti-Inflammatory	Anti-Proteomic	−	4	[168,169]
Pro-Inflammatory	Energy Consumption	+	4	[170,171]
Pro-Inflammatory	Energy Production	+	4	[172,173]
Pro-Inflammatory	Metal Ion	−	3	[174]
Pro-Inflammatory	Metal Ion Chelator	+	1	N/A
Pro-Inflammatory	Anti-Excitotoxicity	−	4	[175,176]
Pro-Inflammatory	Anti-Proteomic	+	1	N/A
Pro-Inflammatory	Pro-Proteomic	−	4	[177,178]
Antioxidants	Energy Consumption	−	3	[179]
Antioxidants	Metal Ion	+	1	N/A
Antioxidants	Metal Ion Chelator	−	1	N/A
Antioxidants	Anti-Excitotoxicity	+	3	[180]
Antioxidants	Pro-Excitotoxicity	+	3	[181]
Antioxidants	Anti-Proteomic	+	4	[182,183]
Pro-Oxidative	Energy Consumption	−	4	[184]
Pro-Oxidative	Metal Ion Chelator	+	3	[185]
Pro-Oxidative	Anti-Excitotoxicity	+	4	[186]
Pro-Oxidative	Pro-Excitotoxicity	+	4	[187,188]
Pro-Oxidative	Anti-Proteomic	−	4	[189,190]
Anti-Proteomic	Energy Consumption	+	3	[191]
Anti-Proteomic	Metal Ion Chelator	+	3	[192]
Anti-Proteomic	Anti-Excitotoxicity	+	3	[193]
Anti-Proteomic	Anti-Inflammatory	+	4	[194,195]
Anti-Proteomic	Pro-Oxidative	−	2	[196]
Pro-Proteomic	Anti-Apoptosis	−	4	[197,198]
Pro-Proteomic	Energy Consumption	−	4	[52,199]
Pro-Proteomic	Energy Production	+	4	[200,201]
Pro-Proteomic	Metal Ion	+	3	[202]
Pro-Proteomic	Metal Ion Chelator	−	3	[203]
Pro-Proteomic	Anti-Excitotoxicity	−	4	[204,205]
Pro-Proteomic	Pro-Excitotoxicity	+	4	[206,207]
Pro-Proteomic	Pro-Inflammatory	+	3	[208]
Pro-Proteomic	Antioxidants	+	3	[209]
Pro-Proteomic	Anti-Proteomic	−	4	[210]
Pro-Proteomic	Pro-Proteomic	+	1	N/A
